# A fixed dose combination of tamsulosin and tadalafil in men with benign prostatic hyperplasia and erectile dysfunction: a prospective, multicenter, phase IV study

**DOI:** 10.1007/s00345-026-06306-3

**Published:** 2026-03-01

**Authors:** Chirag Gupta, Avinash T. S., Dushyant Shamlabhai Pawar, Abhay Khandekar, G. Ravindra Deshmukh, Vinay Kumar, Ajwani Vikky Ramesh, Shah Ishan Hemantkumar, Patankar Suresh Balkrishna, Mandodari Rajurkar, Dipesh Sonawane, Dipak Patil, Pravin Ghadge, Suyog Mehta

**Affiliations:** 1 Department of Urology, Jaipur National University Institute for Medical Science and Research Center, Jaipur, Rajasthan India; 2 Department of Urology, Sparsh Super Specialty Hospital, Bangalore, Karnataka India; 3 Department of Urology, Indus Hospital, Ahmedabad, Gujarat India; 4 Department of Urology, Siddhivinayak Hospital, Ahmedabad, Gujarat India; 5 Department of Urology, Jasleen Hospital, Nagpur, Maharashtra India; 6 Department of Surgery, GSVM Medical College, Kanpur, Uttar Pradesh India; 7 Department of Urology, Anand Multispecialty Hospital, Vadodara, Gujarat India; 8 Department of Urology, Dr. M K Shah Medical College and Research Center, Smt S.M.S. Multispecialty Hospital, Ahmedabad, Gujarat India; 9 Department of Urology, Ace Hospital and Research Center, Pune, Maharashtra India; 10 Medical Affairs and Clinical Research, Sun Pharma Laboratories Limited, Plot 201 B/1, Sun House, Western Express Highway, Goregaon (E), Mumbai, Maharashtra 400063 India

**Keywords:** Benign prostatic hyperplasia, Erectile dysfunction, International prostate symptom score, Lower urinary tract symptoms, Tamsulosin, Tadalafil

## Abstract

**Purpose:**

To evaluate the safety and efficacy of a fixed-dose combination (FDC) of tamsulosin prolonged release (PR) and tadalafil in moderate-severe benign prostatic hyperplasia (BPH) and erectile dysfunction (ED).

**Methods:**

This was a single-arm, phase IV, prospective clinical trial in sexually active men aged 45–75 years with BPH [International Prostate Symptom Score (IPSS) score ≥ 8] and ED [International Index of Erectile Function-Erectile Function (IIEF-EF) score ≤ 25] who were taking tamsulosin 0.4 mg PR and tadalafil 5 mg. Eligible patients received FDC of tamsulosin+tadalafil (0.4 + 5 mg) capsules for 12 weeks. The endpoints included treatment-emergent adverse events (TEAEs), total IPSS, IPSS storage and voiding sub-scores, maximum urinary flow rate (Q_max_), post-void residual (PVR) volume, IIEF-EF (questions 1–5 and 15) score, and IPSS quality of life (QoL) index.

**Results:**

A total of 172 were enrolled. Overall, 12 TEAEs were reported in 10 (5.81%) patients. None of the TEAEs were severe, serious, life-threatening, or required treatment interruption. A statistically significant improvement (*p* < 0.0001) in total IPSS after 4, 8, and 12 weeks was observed. Similar improvements from baseline were also observed in IPSS storage and voiding sub-scores, IPSS QoL, Q_max_, PVR volume, and IIEF-EF (questions 1–5 and 15) score (*p* < 0.05 for each parameter). The proportion of men with normal erectile function significantly increased at week 12 (*p* = 0.0003).

**Conclusion:**

The FDC of tamsulosin and tadalafil was associated with significant improvements in lower urinary tract symptoms and EF over 12 weeks and was well tolerated in Indian men with moderate-severe BPH and ED.

**Trial registration:**

Prospectively registered at Clinical Trials Registry—India on 10th June 2022 [CTRI/2022/06/043152].

## Introduction

Aging causes physical changes in men, including benign prostatic hyperplasia (BPH) and erectile dysfunction (ED) [1]. BPH leads to lower urinary tract symptoms (LUTS), and about three-fourths of men with LUTS/BPH have co-existing ED. As the severity of LUTS increases, the prevalence rate of ED in these patients also increases and ranges from 35 to 95%. Often, ED co-exists with LUTS/BPH and vice versa [2]. The Global Burden of Disease Study 2019 indicated that there were 51.1 million cases of BPH globally in the year 2000, which increased to 94.0 million cases in 2019. The number of BPH cases in India increased by 90.9%, rising from 9.55 million in 2000 to 18.2 million in 2019 [3]. According to the United Nations Population Division, the life expectancy of the Indian population at birth for both sexes combined increased from 41.2 years in 1950 to 72.0 years in 2023. It is expected to rise further to 77.5 years in 2050 [4]. The prevalence of ED and LUTS increases with age and negatively impacts the quality of life (QoL) [5]. As the life expectancy is rising continuously, there is a need for effective treatment strategies for the rising cases of LUTS/BPH and ED [3, 4].

Guidelines by the Urological Society of India and the European Association of Urology recommend α_1_-blockers in men with moderate to severe LUTS [6–8]. The European Association of Urology also recommends the combination of α_1_-blockers and phosphodiesterase-5 inhibitors (PDE5Is) in men with bothersome LUTS, especially in those willing to improve their erectile function [8]. The American Urological Association (2023 guidelines) recommends the use of low-dose tadalafil 5 mg daily with α_1_-blockers for the treatment of BPH/LUTS [9]. α_1_-blockers efficiently reduce BPH symptoms and improve the associated urodynamic parameters of obstruction [10].

Tamsulosin is an α_1_-blocker approved for treating LUTS and significantly improves subjective symptoms [11]. The improvement in international prostate symptom score (IPSS), post-void residual (PVR) volume, and maximum urinary flow rate (Q_max_) with tamsulosin is greater than with the other α_1_-blocker monotherapies for BPH [12].

Currently, the efficacy of PDE5Is in managing LUTS is established. Tadalafil 5 mg, a PDE5I, is approved as a once-daily treatment in patients with LUTS/BPH irrespective of ED [13]. Tadalafil has been demonstrated to reduce the IPSS by 22–37% [1].

Several published studies reporting the efficacy and safety of the combination of tamsulosin 0.4 mg and tadalafil 5 mg in patients with BPH/LUTS irrespective of co-existing ED are available [11, 14, 15]. There is a dearth of data regarding the use of this combination in the Indian population with moderate to severe BPH and ED. Therefore, in this single-arm, phase IV, prospective, multicenter, interventional clinical trial, we aimed to evaluate the safety and efficacy of this fixed-dose combination (FDC) in men with moderate to severe BPH and ED.

## Patients and methods

### Study design

This was a single-arm, open-label, phase IV, prospective, multicenter, interventional clinical study (prospectively registered at https://ctri.nic.in/Clinicaltrials/pmaindet2.php?EncHid=Njk2NDU=&Enc=&userName=%20%E2%80%94%20CTRI/2022/06/043152).

It was conducted from 23/AUG/2022 to 22/JUL/2023 and enrolled men with BPH and ED at 9 sites across India. The study aimed to determine the safety and efficacy of the FDC of tamsulosin hydrochloride (HCl) prolonged release (PR) 0.4 mg and tadalafil 5 mg capsules in BPH and ED.

### Ethical considerations

The research was conducted in accordance with the Declaration of Helsinki and Good Clinical Practice guidelines. The institutional ethics committee at each site approved the study before study initiation. A written informed consent was obtained from participants prior to screening for the study.

### Key inclusion criteria

Sexually active men aged 45–75 years with (1) BPH and ED and taking both tamsulosin HCl PR 0.4 mg and tadalafil 5 mg concomitantly at screening; (2) total IPSS score ≥ 8 (moderate to severe BPH), Q_max_ between 5 and 15 mL/s (both included) with minimum voided volume of > 125 mL, PVR volume < 300 mL (assessed by ultrasound), and international index of erectile function-erectile function (IIEF-EF) score of ≤ 25 at enrolment were included in this study.

### Key exclusion criteria

Patients with (1) prostate specific antigen > 4 ng/mL; (2) history of neurologic bladder, urethral strictures, urinary tract infections, prostatitis, urologic cancer, and prostatic surgery; (3) clinically significant bladder outflow obstruction other than BPH (calculi, tumor or stricture); (4) any other bladder or urinary tract conditions, which could affect LUTS; (5) use of any 5-alpha reductase inhibitor, drugs with androgenic or anti-androgenic properties, anabolic steroids, luteinizing hormone-releasing hormone agonists/antagonists or other treatments affecting prostate volume, within 3 months prior to screening visit were excluded from the study.

Patients with diabetes mellitus, moderate to severe renal or hepatic impairment, or any other clinically significant disorder that, in the opinion of the study doctor, would interfere with the ability to understand and comply with the study requirements were also excluded.

### Study treatment and assessments

During the study, eligible patients were enrolled and administered with FDC of tamsulosin HCl PR 0.4 mg and tadalafil 5 mg capsules (Contiflo T Capsule PR^®^, manufactured by Sun Pharma) orally once daily, half an hour after dinner, for 12 weeks.

The duration of screening and washout periods was up to 7 and 28 days, respectively, and the baseline/enrolment visit was considered Day 1. Baseline assessments were done before administration of the first dose. Patients were followed up every 4 weeks for study assessments, and week 12 was the end of the study visit.

### Study outcomes

The primary outcome measure was safety assessment based on the proportion of patients experiencing treatment-emergent adverse events (TEAEs). Adverse events (AEs) were defined and coded using the Medical Dictionary for Regulatory Activities (MedDRA) version 27.0 and graded for severity according to the Common Terminology Criteria for AEs (CTCAE) version 5.0. AEs were assessed systematically at each study visit until the end of the follow-up period.

The secondary outcome measures were change from baseline to weeks 4, 8, and 12 in total IPSS, IPSS storage (irritative) and voiding (obstructive) sub-scores, Q_max_, PVR volume, and IIEF-EF (questions 1–5 and 15) score. Additional secondary outcome measures included change in IPSS QoL index from baseline to week 12 and proportion of patients demonstrating normal erectile function (IIEF-EF score > 26) at the end of week 12.

### Sample size and statistical analysis

Approximately 173 patients were required. A proportion test using normal approximation with a 5% two-sided significance level, assuming a 4.5% margin of error and an incidence rate for AEs of 9%, required a sample size of 156 patients. With an attrition rate of 10% due to various reasons, a sample size of 173 patients was required.

Baseline demographics were evaluated on the intention-to-treat (ITT) population, which included all patients who were enrolled in the study. Safety analysis was conducted on the safety population, which included patients who received at least one dose of the treatment. The proportion of participants in the safety analysis was summarized using counts and percentages. Efficacy analysis was conducted on the modified ITT (mITT), which included patients who met all eligibility criteria, received at least one dose, and attended at least one post-baseline assessment. Change from baseline at the end of follow-up visits was analyzed using the Wilcoxon signed-rank test. Statistical Analysis System version 9.4 was used for statistical analysis, and *p* < 0.05 indicated statistical significance.

## Results

**Fig. 1:**
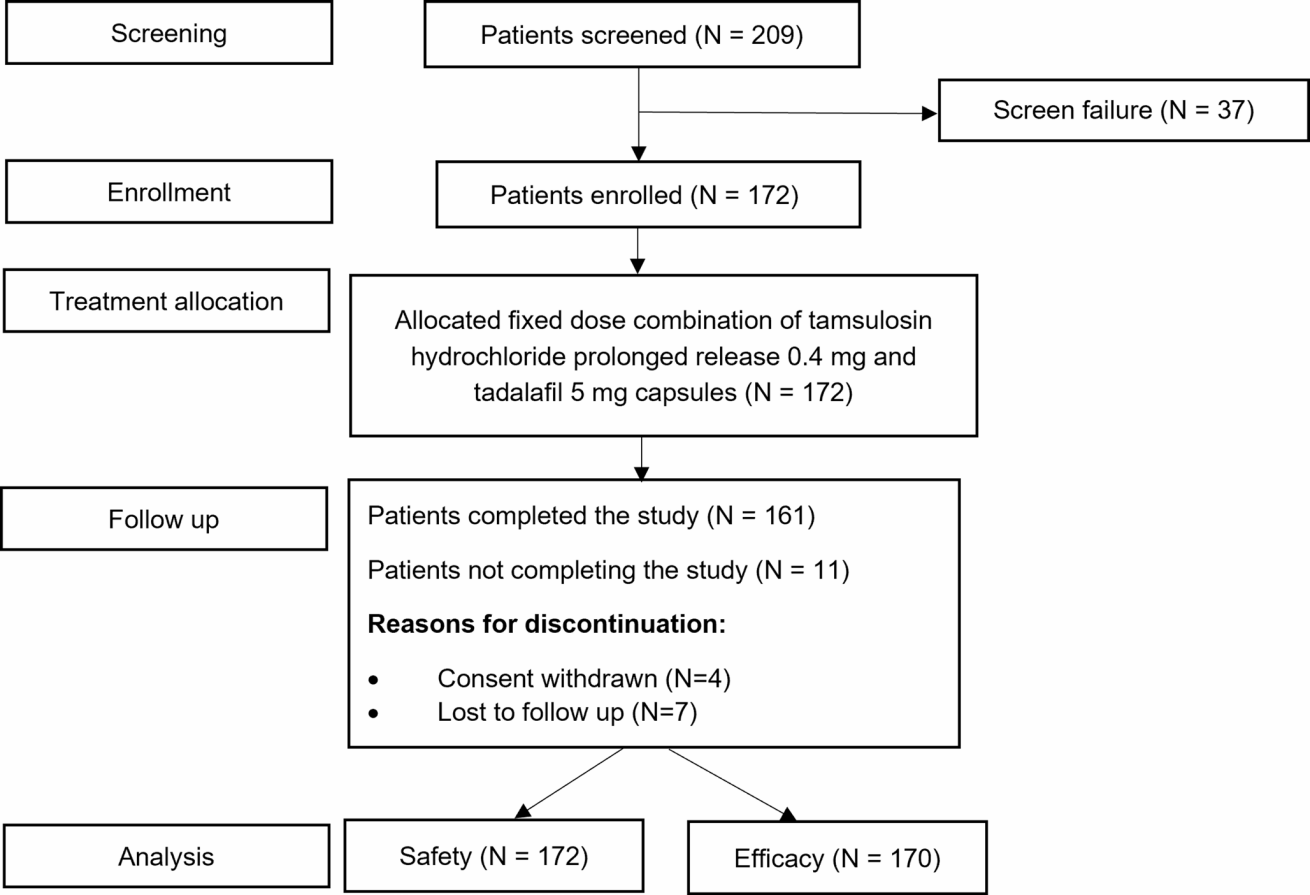
Participant flow through the study

A total of 209 patients were screened for eligibility, and 172 were included in the study, out of which 161 patients completed the study. Participant flow through the study is demonstrated in Fig. 1. Overall, 161 of the 172 enrolled patients demonstrated treatment compliance greater than 90%, indicating a good level of adherence to the study treatment.

Baseline demographics and clinical characteristics are provided in Table 1. The commonly reported prior medications included domperidone, rabeprazole, lactulose, and *Plantago ovata* husk. The most frequently used concomitant medications were paracetamol and azithromycin.

**Table 1:** Baseline demographics and clinical characteristics

Baseline demographics (ITT population, *N* = 172^$^) and clinical characteristics (mITT population, *N* = 170^#^)	Fixed dose combination of tamsulosin hydrochloride prolonged release 0.4 mg and tadalafil 5 mg capsules
Age, years^$^	56.20 (8.04)
Height, cm^$^	164.78 (7.08)
Weight, kg^$^	68.06 (9.55)
Total IPSS^#^	19.60 (5.84)
IPSS storage (irritative) sub-score^#^	8.50 (2.69)
IPSS voiding (obstructive) sub-score^#^	11.10 (3.67)
IPSS QoL Index^#^	4.40 (0.80)
Q_max_, mL/sec^#^	10.00 (2.66)
PVR volume, mL^#^	47.47 (53.01)
IIEF-EF (questions 1–5 and 15) score^#^	13.60 (5.12)
Proportion of patients with normal erectile function (IIEF-EF score > 26), n (%)^#^	0.00 (0.00)

Data presented as mean (SD), unless otherwise specified

*IIEF-EF* International index of erectile function-Erectile function, *IPSS* International prostate symptom score, *ITT* Intention-to-treat, *mITT* Modified intention-to-treat, *PVR* Post-void residual, *Qmax* Maximum urinary flow rate, *QoL* Quality of life, *SD* standard deviation

^$^ITT population

^#^mITT population

### Safety results

Overall, 12 TEAEs were reported in 10 (5.81%) patients. Of the 12 TEAEs, 2 (hyperleukocytosis and back pain) were mild and 10 were moderate in intensity. All the reported TEAEs were unlikely to be related to the study treatment and were resolved without any sequelae at the end of the study. None of the TEAEs were severe, serious, life-threatening, or required treatment interruption, discontinuation, or dose reduction.

The most frequently reported TEAE was nasopharyngitis, occurring in 4 (2.33%) patients. Other TEAEs included headache (2 patients, 1.16%), pain (1 patient, 0.58%), pyrexia (1 patient, 0.58%), vomiting (1 patient, 0.58%), back pain (1 patient, 0.58%), cough (1 patient, 0.58%), and hyperleukocytosis (1 patient, 0.58%).

### Efficacy results

After 12 weeks of treatment, total IPSS demonstrated a statistically significant improvement (*p* < 0.0001). Similar significant improvements in total IPSS were observed at weeks 4 and 8 (*p* < 0.0001, each). Statistically significant improvements in efficacy from baseline were observed (Table 2).

**Table 2:** Change in efficacy parameters from baseline to 12 weeks in patients treated with tamsulosin hydrochloride prolonged release 0.4 mg and tadalafil 5 mg

Efficacy parameters	Baseline (*N* = 170)	Week 4 (*N* = 170)	Week 8 (*N* = 170)	Week 12 (*N* = 170)
Total IPSS	19.60 (5.84)	15.40 (5.84)	13.80 (5.40)	12.60 (5.33)
Change from baseline		− 4.20 (5.85)^*^	− 5.80 (5.82)^*^	− 7.00 (6.09)^*^
IPSS storage (irritative) sub-score	8.50 (2.69)	6.80 (2.69)	6.10 (2.46)	5.60 (2.34)
Change from baseline		− 1.60 (2.41)^*^	− 2.30 (2.32)^*^	− 2.90 (2.48)^*^
IPSS voiding (obstructive) sub-score	11.10 (3.67)	8.50 (3.47)	7.70 (3.25)	7.00 (3.23)
Change from baseline		− 2.60 (3.85)^*^	− 3.40 (4.02)^*^	− 4.10 (4.13)^*^
IPSS QoL Index	4.40 (0.80)	3.50 (1.01)	3.20 (1.05)	2.80 (1.09)
Change from baseline		− 0.80 (1.04)^*^	− 1.20 (1.12)^*^	− 1.60 (1.21)^*^
Q_max_, mL/sec	10.00 (2.66)	12.70 (7.25)	12.40 (6.88)	13.20 (8.24)
Change from baseline		2.65 (6.99)^*^	2.41 (6.59)^*^	3.18 (7.91)^*^
PVR volume, mL	47.47 (53.01)	39.05 (47.44)	36.32 (45.14)	39.22 (50.89)
Change from baseline		− 8.40 (41.14)^*^	− 11.10 (44.36)^*^	− 8.20 (42.37)^*^
IIEF-EF (questions 1–5 and 15) score	13.60 (5.12)	15.30 (6.03)	15.70 (6.10)	15.70 (6.74)
Change from baseline		1.60 (4.31)^*^	2.00 (5.76)^*^	2.00 (6.97)^$^
Proportion of patients with normal erectile function (IIEF-EF score > 26), n (%)^@^	0.00 (0.00)	NE	NE	11 (6.83)^**^

Data presented as mean (SD), unless otherwise specified

*IIEF-EF* International index of erectile function-Erectile function, *IPSS* International prostate symptom score, *NE* Not evaluated, *PVR* Post−void residual, *Qmax* Maximum urinary flow rate, *QoL* Quality of life, *SD* Standard deviation

*p<0.0001; $*p*=0.0009; **p=0.0003; @total number of patients evaluated at week 12 was 161

## Discussion

To the best of our knowledge, this is the first study to evaluate the efficacy and safety of the FDC of tamsulosin 0.4 mg and tadalafil 5 mg in the Indian population with moderate to severe BPH and ED. Our findings demonstrate a statistically significant improvement (*p* < 0.0001) in total IPSS after 4, 8, and 12 weeks of treatment with the FDC of tamsulosin 0.4 mg and tadalafil 5 mg. Similar improvements from baseline were also observed in IPSS storage and voiding sub-scores, IPSS QoL, Q_max_, PVR volume, and IIEF-EF (questions 1–5 and 15) score (*p* < 0.0001 for each parameter at each visit and *p* = 0.0009 for IIEF-EF at Week 12). The proportion of patients with normal erectile function increased by 6.83% (*p* = 0.0003) from baseline to week 12.

Our findings are consistent with several international studies supporting the efficacy and safety of the tamsulosin and tadalafil combination in patients with BPH and ED. Various systematic reviews and meta-analyses have shown significant improvements in LUTS, IIEF scores, IPSS, and Q_max_ with combination therapy, irrespective of ED status [11, 14–17]. A meta-analysis conducted by Sun K et al. included eight studies enrolling 1144 participants with LUTS and ED related to BPH. Among these studies, seven evaluated the combination of tamsulosin and tadalafil, while one study evaluated the combination of tamsulosin and sildenafil. The comparator groups were tamsulosin or tadalafil monotherapy. The results indicated that the combination group performed significantly better in terms of the IPSS and Q_max_ compared to the monotherapy groups. Additionally, the combination group demonstrated greater efficacy in relation to the IIEF when compared to the tamsulosin group [17]. Several individual studies assessing the efficacy and safety of the combination of tamsulosin 0.4 mg and tadalafil 5 mg in patients with BPH or LUTS further corroborate these results [1, 13, 18–24].

A meta-analysis of 12 randomized controlled trials on PDE5Is in patients with LUTS due to BPH suggested that combination therapy with α_1_-blockers, particularly with tamsulosin, allows achieving an additional significant improvement of IPSS, IIEF-EF score, and Q_max_ as compared to monotherapy with α_1_-blockers [16].

In the present study, patients were enrolled based on an IIEF-EF score ≤ 25, with a mean baseline IIEF-EF score of 13.60, indicating that the study population predominantly comprised patients with mild to moderate ED. This baseline characteristic may partly explain the relatively modest improvement observed in IIEF-EF scores over 12 weeks. However, the exact contribution of baseline ED severity to the magnitude of change in erectile function outcomes could not be established. Additionally, tamsulosin 0.4 mg is known to be associated with ejaculatory dysfunction, with a reported incidence of 8.4% [25]. However, ejaculatory dysfunction was not reported as an AE in this study. As ejaculatory function was not a predefined or separately assessed outcome and no ejaculatory-specific instruments were used, the absence of reported cases should be interpreted cautiously. It cannot be used to conclude the effect of the FDC on ejaculatory function.

A published network meta-analysis using p-score rankings indicated that tamsulosin 0.4 mg had the highest probability of being an effective treatment for improving IPSS and Q_max_ compared to doxazosin 8 mg and naftopidil 50 mg. Tamsulosin 0.4 mg also demonstrated the highest probability of being an effective treatment for improving PVR volume; however, the effectiveness was similar to naftopidil [12]. Silodosin is a newer α_1A_ receptor blocker with greater selectivity; however, the outcome of a 2-year hospital-based prospective randomized study indicated that tamsulosin and silodosin are equally effective and safe in managing LUTS due to BPH [26].

Beyond clinical efficacy, the findings in the present study have practical implications for treatment strategies in real-world settings. In the Indian context, FDCs offer additional advantages, particularly in improving adherence through reduced pill burden, a significant consideration for patients requiring long-term treatment [27]. This becomes especially relevant in managing chronic conditions such as BPH and ED, where sustained therapeutic outcomes depend heavily on patient compliance. As a phase IV (post-marketing) regulatory study, the primary objective was to generate safety data under real-world conditions in Indian patients, in line with the approved prescribing information. Accordingly, the study design closely resembled routine clinical practice rather than an interventional trial setting. Moreover, most published evidence to date is based on free-drug combinations or concomitant use of tamsulosin and tadalafil. In contrast, the present study evaluates an FDC in the Indian population, which represents a unique and clinically relevant aspect of this study.

Despite the strengths of the study, there were certain limitations that should be acknowledged. First, the single-arm design precludes direct comparison with a placebo or active control. The absence of a control group prevents definitive conclusions about superiority or causality. Second, while the short-term study duration of 12 weeks allowed for the evaluation of safety and efficacy, it remains unclear whether these benefits persist over a longer period. The study did not evaluate sustained efficacy or the potential for AEs due to long-term treatment with this FDC. Further randomized, double blind, active-controlled studies for a longer duration must be conducted to confirm the findings of this study. Third, median prostate volume was not assessed at baseline, which limits the ability to correlate treatment outcomes with prostate size. Fourth, while erectile function was assessed using the IIEF-EF score, ejaculatory function was not a predefined or separately evaluated endpoint in this study. No ejaculatory-specific validated questionnaires or structured assessments were included, and AE reporting was the only source of information regarding ejaculatory dysfunction. Therefore, the study was not designed to detect changes in ejaculatory function or to evaluate the effects of the FDC on ejaculatory outcomes. Additionally, as this was a phase IV study conducted in a real-world setting, special investigations such as hormonal assessments were not performed at screening; however, patients with a known history of relevant hormonal and psychogenic disorders were excluded.

## Conclusion

The FDC of tamsulosin HCl PR 0.4 mg and tadalafil 5 mg demonstrated significant improvements in LUTS and erectile function over a 12-week period in Indian patients with moderate to severe BPH and ED. The treatment was generally well tolerated, with a low incidence of TEAEs, all of which were mild to moderate in intensity and resolved without sequelae. The findings of this phase IV, single-arm study support the short-term safety and efficacy of this FDC in the management of BPH-associated LUTS and ED.

## Data Availability

The trial protocol and statistical analysis plan are available from the corresponding author upon reasonable request. Individual participant data that have been de-identified, along with the associated data dictionary, statistical code, and other relevant study materials, can also be provided by the corresponding author, subject to approval and in accordance with institutional and ethical guidelines.
